# Assessment of Imaging Mass Cytometry (IMC) as a Tool to Characterize Circulating Tumor Cells (CTCs) in Preclinical Mouse Models

**DOI:** 10.64898/2025.12.18.695262

**Published:** 2025-12-22

**Authors:** Milind Pore, Kuppusamy Balamurugan, Abigail Atkinson, Devynn Breen, Paul Mallory, Ashley Cardamone, Lois McKennett, Christine Newkirk, Shikha Sharan, William Bocik, Esta Sterneck

**Affiliations:** 1 Imaging Mass Cytometry Laboratory, Frederick National Laboratory for Cancer Research, Leidos Biomedical Research, Inc., National Cancer Institute, Frederick, MD, USA; 2 Cancer Innovation Laboratory, Center for Cancer Research, National Cancer Institute, Frederick, MD, USA; 3 Laboratory Animal Sciences Program, Frederick National Laboratory for Cancer Research, Leidos Biomedical Research, Inc., National Cancer Institute, Frederick, MD, USA

**Keywords:** imaging mass cytometry, imaging-proteomics, circulating tumor cells, liquid biopsy, xenograft

## Abstract

Circulating tumor cells (CTCs), and especially CTC-clusters, are linked to poor prognosis and may reveal mechanisms of metastasis and treatment resistance. Therefore, developing unbiased methods for the functional characterization of CTCs in liquid biopsies is an urgent need. Here, we present an evaluation of multiplex imaging mass cytometry (IMC) to analyze CTCs in mice with human xenograft tumors. In a single-step process, IMC uses metal-labeled antibodies to simultaneously detect a large number of proteins/modifications within minimally manipulated small volumes of blood from the tail vein or heart. We used breast cancer cell lines and a patient-derived xenograft (PDX) to assess antibodies for cross-species interpretation. Along with manual verification, HALO-AI-based cell segmentation was used to identify CTCs and quantify markers. Despite some limitations regarding human-specificity, this technology can be used to investigate the effect of genetic and pharmacological interventions on the properties of single and cluster CTCs in tumor-bearing mice.

## Introduction

Metastasis is in part due to circulating tumor cells (CTCs), especially CTC clusters, which are associated with poor prognosis and treatment resistance [1–3]. CTCs increase in numbers after biopsy, surgery, and during neo-adjuvant treatment [4] leading to their exploration as therapeutic targets[5]. CTCs can be assessed with liquid biopsies, which hold immense promise for cancer patients by enabling non-invasive, real-time monitoring of tumor dynamics [6].While many advancements in techniques to isolate and/or enumerate CTCs have been made, investigations of biological mechanisms are very challenging due to their scarcity. Almost all CTC detection methods rely on two steps, first to enrich the CTCs from the millions of blood cells and then specifically isolate/ detect the CTCs based on protein marker expression. In addition, the enrichment and isolation techniques that are required for the study of CTCs in clinical samples invariably expose the cells to physical manipulations and stresses which may alter characteristics of the cell [7, 8]. Because of the high sensitivity of nucleic acid-based methodologies, most characterizations have been conducted at the level of genomics and transcriptomics compared to proteomics or metabolomics [9], although proteins are the most functionally relevant entities. Detection of proteins is especially relevant for insights into protein modifications or subcellular localization. Lastly, while CTCs from patients may provide valuable correlative information, only preclinical mouse models permit genetic and pharmacological manipulations that will lead to mechanistic insights and facilitate the development of novel therapeutics [10].

Multiplex imaging mass cytometry (IMC) of metal-labeled antibodies allows for the simultaneous detection of more than 40 proteins in situ in an exclusive single-step process [11]. Though IMC is mostly employed for spatial analysis of single cells in tissues [12, 13], a small number of studies have applied the technology to CTCs from patients [14, 15]. Here, we evaluated a panel of 31 metal-labeled antibodies for simultaneous detection and characterization of specifically human breast cancer CTCs in small volumes of mouse blood in a single step process without prior enrichment of CTCs. Antibodies were validated with cell lines and cell line spiked mouse blood samples. The cell line derived xenograft models were used to detect and characterize the CTCs. These CTCs were calculated manually and with HALO-AI-based automated pipeline. In the HALO analysis, a cell segmentation classifier was trained, and single-cell marker quantification and phenotyping was performed.

The panel presented here can be expanded with additional antibodies suitable for specific research questions and as an unbiased tool to assess CTC protein biology. Developing this tool for the detection and functional characterization of single CTCs versus CTC clusters in pre-clinical models will shed light on the biology of this elusive but critical cancer cell population, and lead toward a better assessment of the efficacy and clinical utility of therapeutic drugs [16].

## Materials and Methods

### Cell lines and culture:

MCF-7 cells were obtained from ATCC; SUM149 and SUM159 cells originated from Asterand Bioscience (New York, USA). IBC-3 cells were kindly provided by Dr. Junichi Kurebayashi (Kawasaki Medical School) through Dr. Wendy A. Woodward (MDACC) and MDA-MB-231-LM2-GFP-Luc cells (termed (MB-231-LM2) by Dr. Joan Massague. Cell lines were authenticated in 2022 and 2025 by GenePrint^®^10 (Promega, Wisconsin, USA), and *Mycoplasma* tested by qPCR. Cells were cultured in a 5% CO_2_ incubator at 37°C in media with antibiotics as follows: MCF-7, MDA-MB-231-LM2, and MDA-MB-468 cells were in Dulbecco’s Modified Eagle’s Medium, MCF-7 in addition with 1 mM sodium pyruvate; SUM159 in RPMI with 2 mM glutamine, 10 mM HEPES, 1 mM sodium pyruvate, 1X nonessential amino acids (GIBCO, #11140–050) and 55 mM β-mercaptoethanol (GIBCO, #21985–023); SUM149, and IBC-3 in Ham’s F-12 media (GIBCO, #31765092) with 1 μg/ml hydrocortisone and 5 μg/ml Insulin; Fetal bovine serum (FBS) was added at 10% except for SUM159 (5%). Cell culture grade chemicals were from Sigma Aldrich unless indicated otherwise. As indicated, 5×10^4^ cells were seeded on Millicell EZ Slides (Millipore, cat number PEZGS0816) and analyzed after 24–48 h.

### Western blotting analysis:

Whole cell extracts were prepared and Western blotting performed as described [14] with primary antibodies from Cell Signaling Technology (Pan-keratin #4545T; EpCAM #93790T; Vimentin #5741S), and Santa Cruz Biotechnology (GAPDH #sc32233).

### Xenograft mouse models:

The breast cancer PDX BCM-5471 originated from the PDX Core of the Baylor College of Medicine [17] and propagated in NOD/SCID/Il2rg−/− mice essentially as described [17]. PDX fragments or 2×10^6^ cells of the indicated cell lines were implanted into the inguinal fat pads of 9-17-week-old female mice. Tumor volumes were calculated as V = (W(2) × L)/2. When tumors reached up to 2000 mm^3^, about 100 μl blood was collected from the tail vein and/or by cardiac puncture.

NCI-Frederick is accredited by the Association for Assessment and Accreditation of Laboratory Animal Care International (AALACi) and follows the Public Health Service Policy for the Care and Use of Laboratory. Animal care was provided in accordance with the procedures outlined in the Guide for the Care and Use of Laboratory Animals (National Academies Press, 2011) including those pertaining to studies of neoplasia (National Research Council, 1996). All experiments were conducted under protocols approved by the IACUC at NCI-Frederick.

### Blood collection and preparation for imaging analysis:

Peripheral blood was collected from the tail vein into BD Microtainer tubes with K2EDTA (BD, REF 365974). For spiking, about 10^4^ tumor cells in 10 ul were added to 100 ul blood before red cell lysis with 2 ml ACK lysis buffer (Lonza, #BP10–548E) followed by centrifugation at 1500 rpm for 5 min at 4℃. Cells were washed once with 2 ml of ACK lysis buffer and twice with PBS. For IMC analysis, cells were suspended in 50 μl PBS by gentle pipetting 2–3 times using 20–200 microliter tips, applied to 3-well adhesion slides (Superior Marienfeld#0900100 &0906100) and allowed to be attached on glass surface for 1h at 37℃ in a dry oven.

### Antibody-metal labeling and immunocytochemistry:

Antibodies compatible with immunocytochemistry that were BSA-free and at 100 μg quantity with at least 0.5 mg/ml concentration were purchased from different vendors (Table S1). Metal labeling of antibodies was performed using Maxpar^®^ X8 Multimetal Labeling Kit-40 Rxn (201300) as described by the manufacturer’s protocol (Standard BioTools). Cells on EZ slides were fixed with 2% paraformaldehyde for 20 min, followed by permeabilization with 0.1% Triton X for 10 min. Slides were blocked with 3% BSA in PBS for 1 h and stained with a mixture of metal-labeled antibodies at different dilutions (Table S1) overnight at 4°C. Slides were washed with PBS and counterstained with Cell‐ID^™^ Ir191/193 genomic DNA intercalator (Standard BioTools, #201192B) at 1:500 dilution. Cells were washed with PBS followed by quick dip in ddH2O to remove the salts. Slides were dried and ablated with the Hyperion imaging system.

### Imaging mass cytometry:

Data were acquired by laser ablation with a Hyperion XTi Imaging System (Standard BioTools). The instrument was tuned with 3-element tuning slides provided by the manufacturer prior to sample acquisitions. A minimum dual count of Lu 800, transient crosstalk <25% and resolution (first and second mass) >400 criteria were used to successfully pass the tuning of an instrument. For stage one and two, at least three ROIs of 1500*1500 μm size were ablated. For stage three, with detection of CTCs from xenograft and PDX models, the panorama was generated of smeared cells and the ROIs of 1500*1500 μm size were created to cover all the cells from the 3-well slides. The data obtained in .mcd format was uploaded on MCD viewer software (Standard BioTools) to check the staining and manually identify the CTCs. The CTCs were defined as DNA-IR+, CD45- and Pan-keratin+ cells. Pilot experiments had been performed on two earlier versions of Hyperion imaging system (Hyperion and Hyperion+) with similar results.

### Nuclear segmentation and automated CTC counting:

StarDist, Cellpose, and HALO were used for nuclear segmentation. In QuPath, segmentation was performed with the pre-trained DSB2018 model in StarDist, which uses star-convex object detection [18, 19]. Additionally, segmentation using the pre-trained cyto3 model in Cellpose, a generalist segmentation algorithm, was also assessed in QuPath [20, 21]. The third method utilized the HALO Image Analysis platform (Indica Labs), which uses convolutional neural networks for cell level segmentation. Single cell quantification and CTC phenotyping was performed using the HALO HighPlex FL module v4.2.14 with the CTC phenotype defined to identify DNA3^+^Pan-keratin^+^CD45^−^ cells.

## Results

**Figure 1:**
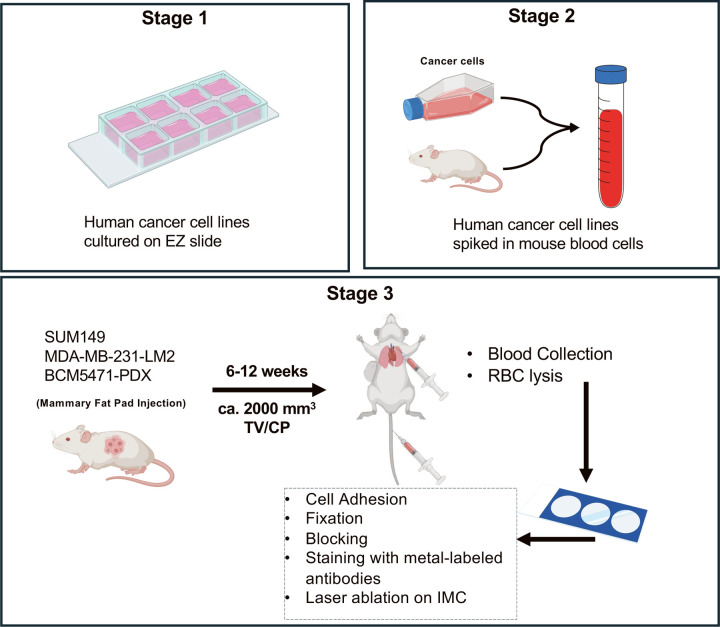
Schematic illustration of the experimental steps toward IMC analysis of CTCs (Figure created in part in BioRender.com).

To assess the performance of specific antibodies for the evaluation of protein expression in human CTCs, we utilized three different experimental approaches as illustrated in Figure 1. Stage 1 evaluated the antibody signals in cell lines cultured on Millicell EZ slides. In stage 2, cell lines grown in conventional cell culture dishes were spiked into control mouse blood to assess staining performance under blood matrix conditions. These samples were spotted onto 3-well adhesion slides as was each component alone as control. Finally, we generated xenograft models by implanting cell lines or a PDX model into mice, and processed blood samples collected via tail vein and/or cardiac puncture for CTC detection and characterization.

**Figure 2.**
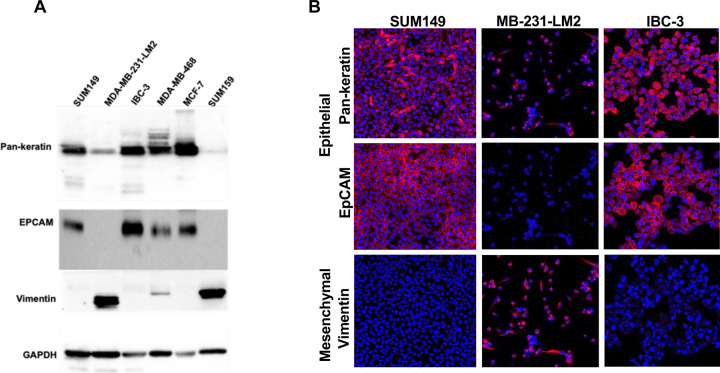
Comparison of Western and IMC analysis of specific antibodies. A). Western blotting analysis of the indicated proteins in whole cell extracts of a panel of breast cancer cell lines. B) Imaging mass cytometry images of proteins in a subset of cell lines grown on EZ slides. The same primary antibodies were used as in panel A, though labelled with unique metals showing red pseudo color (nuclei are shown in blue).

To select suitable cell lines, we first assessed the expression of the epithelial molecule EpCAM and the mesenchymal protein vimentin across a panel of breast cancer cell lines by Western blot analysis (Figure 2A). In addition, we analyzed the reactivity of a Pan-keratin antibody, which produced most signal in epithelial cell lines and, as expected [22], and weak but detectable signals in mesenchymal tumor cells. One mesenchymal (MB-231-LM2) and two epithelial (IBC-3, SUM149) cell lines were chosen to culture directly on glass chambered slides. Three days later, the slides were processed for metal-labeled antibody staining followed by laser ablation on the Hyperion imaging system. EpCAM and vimentin clearly distinguished the three cell lines. IBC-3 and MB-231-LM2 cells showed distinct subcellular abundance and distribution of Pan-keratin with SUM149 presenting a mixed phenotype (Figure 2B). These data suggested that the Pan-keratin antibody may be useful to identify most of these cells irrespective of epithelial/mesenchymal phenotype.

**Figure 3.**
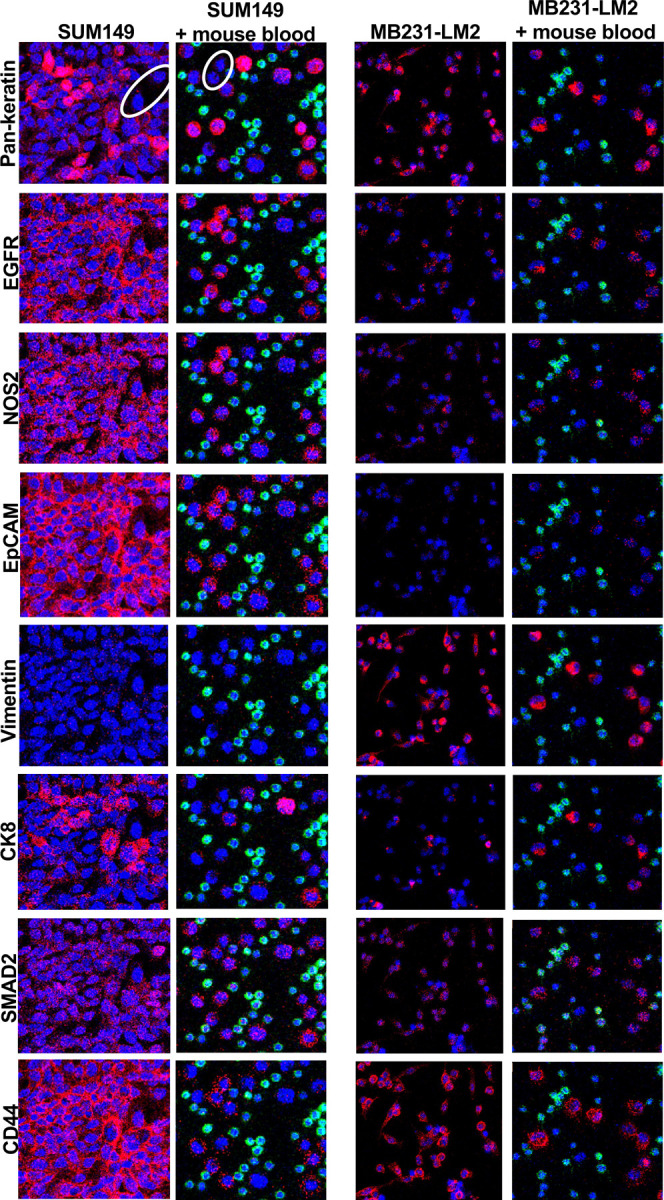
Comparison of IMC signals by specific antibodies in cell lines with and without admixed mouse blood. SUM149 (Left) and MB-231-LM2 (right) cells were spotted directly onto slides or first spiked into mouse blood as indicated. Nuclei are shown in blue and murine immune cells are identified in green by anti-CD45 staining.

**Figure 4.**
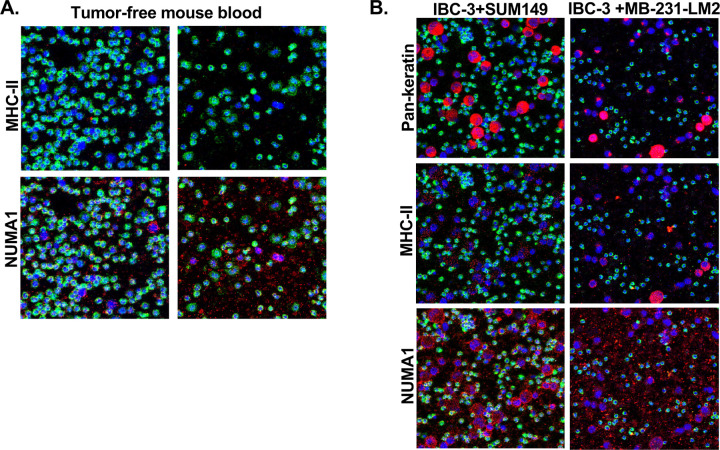
Comparison of human-specific antibodies against MHC-II and NUMA1. (A) IMC analysis of antibodies against MHC-II and NUMA1 in tumor-free mouse blood, and (B) mouse blood spiked the indicated mixture of human cell lines.

Based on these results, we proceeded with SUM149 and MB-231-LM2 cells grown on Millicell EZ slides to assess a panel of metal-labeled antibodies (Stage 1). Next, we spiked these cell lines into mouse blood before spotting onto adhesion slides (Stage 2). We pre-determined that plating 100 μl of nucleated cells from mouse blood on 15×15 mm wells provided sufficient spacing without cell crowding. Stage 2 allowed determination of cross reaction of antibodies with mouse cells as mouse blood without spiking was used as negative control (Figures 3 and S1–3). All SUM149 cells expressed EpCAM but not vimentin and MB-231-LM2 vice versa (Figure 3). Most other markers showed some degree of variability of expression within the spiked cells in each cell line. Interestingly, both panels of SUM149 showed Pan-keratin negative cells (Figure 3, white circles), indicating that this is not a universal marker. Overall signals were higher in stage 1 compared to the stage 2 slides, which could reflect biological differences due to different growth substrates or be due to the processing of cells for spiking. Out of the 31 metal-labeled antibodies tested, 12 showed satisfactory signals in both Stage 1 and Stage 2 (EGFR, NOS2, EpCAM, Vimentin, CK8, Fibronectin, CK18, SMAD2, Cleaved PARP1, Pan-keratin, Cleaved Caspase-3, CD44, plus CD45). Only one of two tested antibodies against E-cadherin (Table S1) gave reliable results in identifying the epithelial IBC-3 cells but not the mesenchymal MB-231-LM2 cells (Figure S3). Several other tested antibodies did not produce satisfactory signals in our hands including one against specifically human Na/K-ATPase α1 protein (Table S1). To not only rely on CD45-negativity for the identification of human cells, we tested two human-specific antibodies against ubiquitously expressed proteins, NUMA1 and MHC-II in a mix of MB-231-LM2 and IBC-3 cells. Anti-NUMA1 showed unspecific staining and high background in mouse blood cells. Anti-MHC-II reacted with all CD45-negative cells, albeit with a high dynamic range, and did not stain any mouse blood cells (Figures 4A–B). We concluded that anti-MHC-II can be used to assist with the identification of human cells at least in some model cell lines.

**Figure 5.**
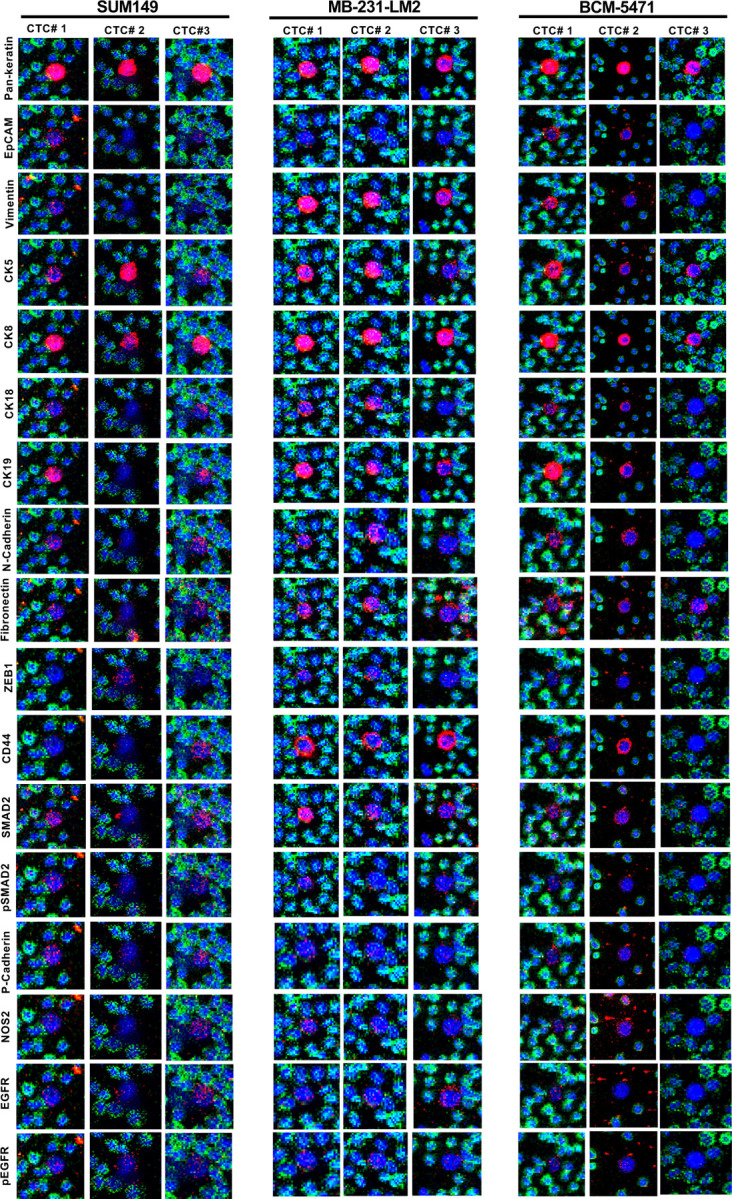
Comparison of IMC results across CTCs and mouse models. IMC images of antibodies against the indicated proteins in three different CTCs each from mice with SUM149, MB-231-LM2 or PDX xenograft tumors.

**Figure 6.**
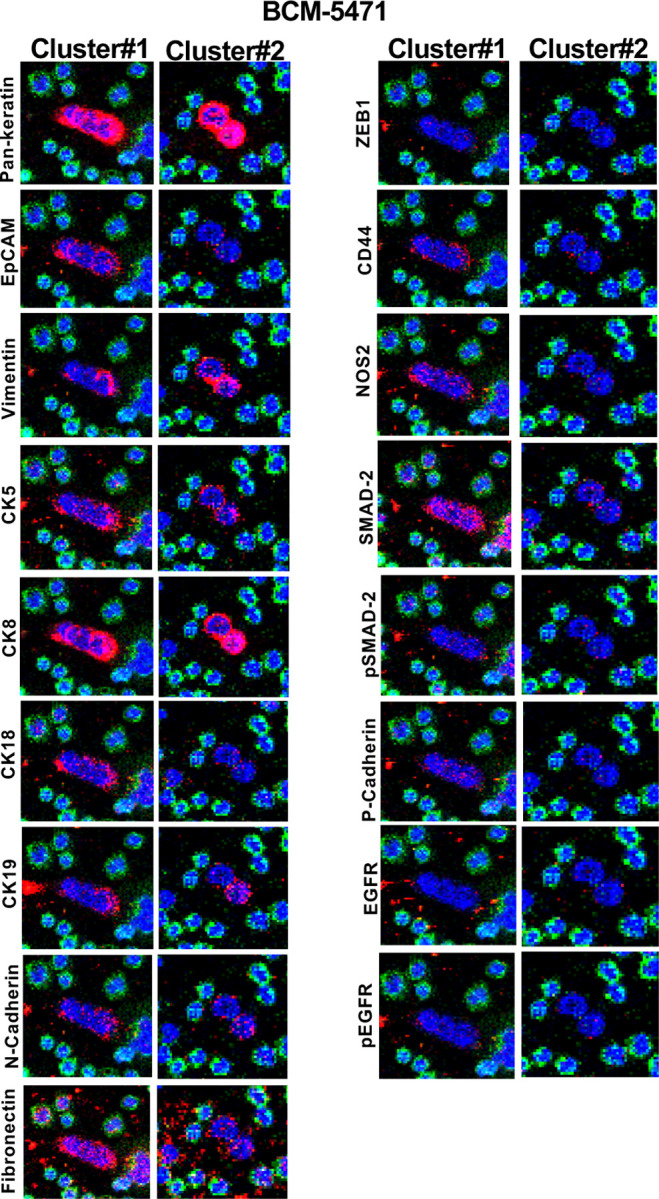
IMC analysis of CTC clusters from BCM-5471 PDX. IMC images of the indicated antibodies in two 2–3 cell clusters of CTCs from mice with BCM-5471 PDX tumors.

In Stage 3, we sought to analyze CTCs in blood from preclinical mouse models with tumors derived from the SUM149 and MB-231-LM2 cell lines or the patient-derived xenograft BCM-5471. CTCs were identified manually by presence of a nucleus, Pan-keratin positivity and CD45 negative staining. Table S2 summarizes the frequency of CTCs in blood collected by tail vein and/or cardiac puncture. We observed a trend toward more CTCs in blood collected from the heart, which was however not statistically significant. Figure 5 shows IMC data for three CTCs from each of the three cancer models. Interestingly, many markers such as CK5, CK18, CK19, ZEB1, P-Cadherin and fibronectin expressions were seen only in Stage 3 CTCs at varying intensities but generated no or weak signals with cells cultured ex vivo (Stage 1 and 2) suggesting that their expression may be induced in vivo. Despite the small number of examples, the data also showed significant variability in expression between CTCs in the same model. For examples SUM149 CTC#2 expressed ZEB1 and higher levels of CK5 than CTC1 and CTC3. In BCM-5471, only CTC#2 showed high levels of CD44 while CTC#3 was negative for most markers. Comparison of two 2–3 cell clusters from BCM-5471showed similarities and contrasts (Figure 6). For example, EpCAM and vimentin showed more signal in Cluster#1 respectively Cluster #2, and cytokeratin 19 was detected in only one cell of each cluster.

To move beyond labor-intensive manual enumeration and evaluation of CTCs, we also developed an automated cell segmentation and CTC detection pipeline. The results from three nuclear segmentation methods (HALO, Cell pose, and StarDist) were compared across multiple images for the three cancer cell models in the study (Figure S4A). In evaluating the accurately identified nuclei from the images surveyed, the HALO AI segmentation performed better than the StarDist and Cellpose pre-trained methods. The StarDist model had difficulty identifying nuclei in dense clusters while Cellpose missed detection of lower intensity nuclei. Moving forward with the HALO AI segmentation, over 2000 nuclei from the IMC images were annotated giving the algorithm substantial ground truth examples to train from. The training of the classifier was validated at every ten thousand annotations to evaluate the peak in its performance. At 40k iterations, the validation of several images showed the most accurate detection and segmentation of the nuclei, and therefore, this trained algorithm was chosen as the segmentation module to construct the analysis module. The acquisition of xenografts and PDX derived slides were ablated on different versions of Hyperion Imaging system including, Hyperion, Hyperion+ and the newest Hyperion XTi. This presented differences in the intensity of the nuclear marker, DNA3, which created challenges in using one consistent segmentation module for all images acquired. To accurately detect nuclei on images with lower signal intensities in this channel, HALO AI default pre-trained segmentation was utilized on several images in the analysis that the HALO custom AI underperformed on.

**Figure 7:**
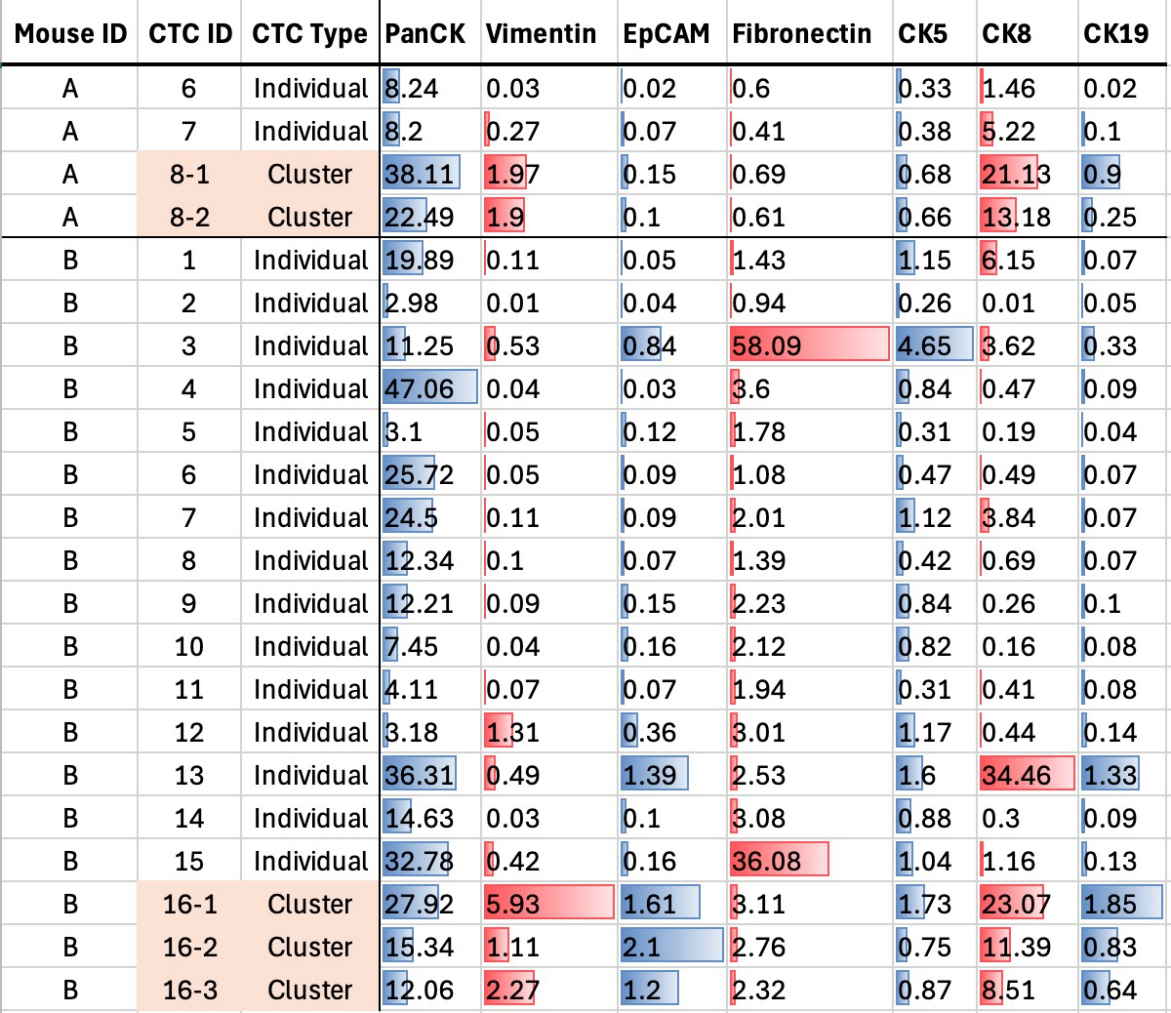
Quantification of IMC signals in single CTCs and CTC clusters. HALO-derived quantification of specific antibody signals for individual cells (CTC ID**#**) in CTC clusters and as single CTCs in blood of BCM-5471 tumor-bearing mice (values are mean marker intensities in arbitrary units).

CTCs were identified by the presence of DNA3 and Pan-keratin, and the absence of the immune cell marker CD45 (Figure S4B). The HALO classification of the CTC phenotype was optimized by performing a visual inspection for Pan-keratin positivity. Due to the signal intensity differences between image sets and instruments, a general survey of cells expressing Pan-keratin was performed first. This preliminary analysis involved broad thresholds with a phenotype defined to include DNA3^+^/Pan-keratin^+^/CD45^−^ cells. The Pan-keratin signal intensity needed to be greater than 1.4 and more than 20 percent completeness, a parameter that requires a set percentage of the cell to be above the minimum positivity threshold, while the CD45 signal needed to be less than 2.0 and 20 percent completeness for the cell to be counted as a CTC. After a review of these preliminary results, thresholds for the DNA3, Pan-keratin, and CD45 were defined for the CTC phenotype per cell model and instrument as outlined in Table S3. Examples of the number of CTC counts with HALO AI cell segmentation classifier in comparison to manual CTC counts is shown in Table S4 and suggested overall concordance. Importantly, HALO AI segmentation enables the quantification of expression differences at the single cell level. To demonstrate this utility, Figure 7 shows examples of quantifications of several signals in single and cluster CTCs from two mice with BCM-5471 tumors with one cluster CTC each. The data again illustrate significant variations between CTCs and can be used to generate hypotheses such as preferential co-expression of vimentin and cytokeratin 8 in cluster CTCs, suggesting a hybrid E/M phenotype which is highly relevant for metastasis [23].

## Discussion

Currently, enumeration of CTCs is the only aspect with clear level of evidence for the utility of CTCs in liquid biopsies for clinical practice. However, the phenotypic characterization of CTCs and biomarker expression for response prediction were considered the most important aspects for future clinical applications in a survey of clinical experts [16]. Here, we have assessed IMC of CTCs in liquid biopsies from preclinical mouse models to provide methodology that can accelerate discovery and broader utility of CTCs in the realm of liquid biopsy research and application.

Fluorescent immunostaining has been used for limited targeted investigations of CTCs, but large-scale proteomics approaches are still lacking [24, 25]. However, the number of markers that can be analyzed simultaneously is limited with this technology and multiplex applications that require many cycles of staining and washing risk losing these rare single cells during analysis. This is where IMC as a single step process is superior. Compared to multiplex fluorescent, IMC requires more expensive instrumentation and lacks signal-amplification by secondary antibodies. The advantage of IMC, however, lies in the single-step process with metal-tagged antibodies of which produce unique non-overlapping spectra. IMC has been explored as a technology to characterize CTCs in patients but has not yet reached wider application [14, 26]. Thus, pre-clinical models carry immense promise to hasten discoveries of translationally meaningful CTC biology.

Our current findings along with those from a prior study [27] demonstrate that tail vein sampling is a viable methodology to monitor CTCs and, thus, suitable for longitudinal monitoring. This contrast a prior report that CTCs were not detectable in blood drawn from the tail vein and suggested cardiac puncture for serial determination [28]. We also established that 100 μl of mouse blood represents a sufficient biopsy volume for CTC detection across the tested models. While enrichment of CTCs may be desirable, each isolation method presents specific strengths and weaknesses, most require expensive equipment, and all lead to cell manipulations that may change cell biology [29]. We propose that cell spotting with minimal processing best preserves the original cellular phenotype.

All CTC research faces the challenge to differentiate tumor cells from normal non-immune cells. In xenograft mouse models, we anticipated that this could be “easily” resolved through human-specific antibodies against ubiquitously expressed proteins. Of three antibodies tested in this study, only MHC-II generated satisfactory specific signals in two human cell lines. None of these antibodies reacted with BCM-5471 CTCs, though, which may be due to its downregulation for immune-evasion [30]. By immunohistochemistry, a polyclonal antibody for NUMA1 proved suitable for the detection of human cells in tissue sections of mice [27, 31]. However, the specific antibody formulation was not suitable for IMC. Therefore, another clone was used but produced too much background. Furthermore, since the CD31 antibody signal tested here was not specific, we could not rule out the possibility of detecting mouse endothelial cells that might co-express Pan-keratin and CD31. For these reasons, we recommend that all antibodies be tested against blood from tumor-free mice for comparison. While more antibodies may be still be tested, genetically introduced protein tags or foreign reporter genes can also be considered as tools for cancer cell specificity.

For validation of antibodies at stages 1 and 2, a variety of control cell lines is recommended because we found that CTCs can induce expression of proteins that were not detected in the same cells cultured ex vivo. In our hands, the mesenchymal MBA-MB-231-LM2 cells still reacted with anti-Pan-keratin. The subcellular distribution was droplet-like as had been described for Rhabdomyosarcoma cells [32] and a hepatocyte clone [33]. Therefore, the % cell coverage had to be adjusted for this cell line. For the automated CTC detection, we recommend starting with low thresholds followed by manual verification of called CTCs. Issues encountered at this step were Pan-keratin positivity “without nucleus” and “hybrid cells” lighting up with markers for CTCs and immune cells. It has been proposed that the latter can be the result of phagocytosis of human material by immune cells, cell fusion, or exchange of proteins by cell-cell contact [34–36]. Interpretation of such cells, which were rare, will depend on their value for a specific research question. Like many CTC studies [37], we relied on Pan-keratin to identify cancer cells. However, some cells were negative for Pan-keratin suggesting that purely mesenchymal CTCs could be missed and vimentin could be included in the initial calling of CTCs.

In conclusion, we show that IMC as a robust single-step methodology for the detection and phenotypic characterization of CTCs in preclinical mouse models. The validated workflow presented here provides a platform for interrogating CTC heterogeneity, cluster formation, and dynamic biomarker expression. Application to the study of CTCs in liquid biopsies of mice can contribute to monitoring the effects of genetic alterations and therapeutic interventions, and advance liquid biopsy applications beyond enumeration toward treatment response prediction and precision oncology.

## Supplementary Material

Supplement 1
