# Supplementary material for: Assessment of Imaging Mass Cytometry (IMC) as a Tool to Characterize Circulating Tumor Cells (CTCs) in Preclinical Mouse Models
Source: bioRxiv. 2025 Dec 22:2025.12.18.695262. Preprint. [Version 1] doi: 10.64898/2025.12.18.695262 (PMC12776163; doi:10.64898/2025.12.18.695262)
Supplement: Supplement 1 [file media-1.pdf]

Supplementary Material

Preprint December 17, 2025

Assessment of Imaging Mass Cytometry (IMC) as a Tool to Characterize Circulating Tumor Cells (CTCs) in Preclinical Mouse Models

Milind Pore[1*#], Kuppusamy Balamurugan[2*], Abigail Atkinson[1], Devynn Breen[1], Paul Mallory[1], Ashley Cardamone[1], Lois McKennett[3], Christine Newkirk[1], Shikha Sharan[2], William Bocik[1] and Esta Sterneck[2#]

[1]Imaging Mass Cytometry Laboratory, Frederick National Laboratory for Cancer Research, Leidos Biomedical Research, Inc., National Cancer Institute, Frederick, MD, USA

[2]Cancer Innovation Laboratory, Center for Cancer Research, National Cancer Institute, Frederick, MD, USA

[3]Laboratory Animal Sciences Program, Frederick National Laboratory for Cancer Research, Leidos Biomedical Research, Inc., National Cancer Institute, Frederick, MD, USA

*equal contributions

# Corresponding authors: milind.pore@nih.gov, esta.sterneck@nih.gov

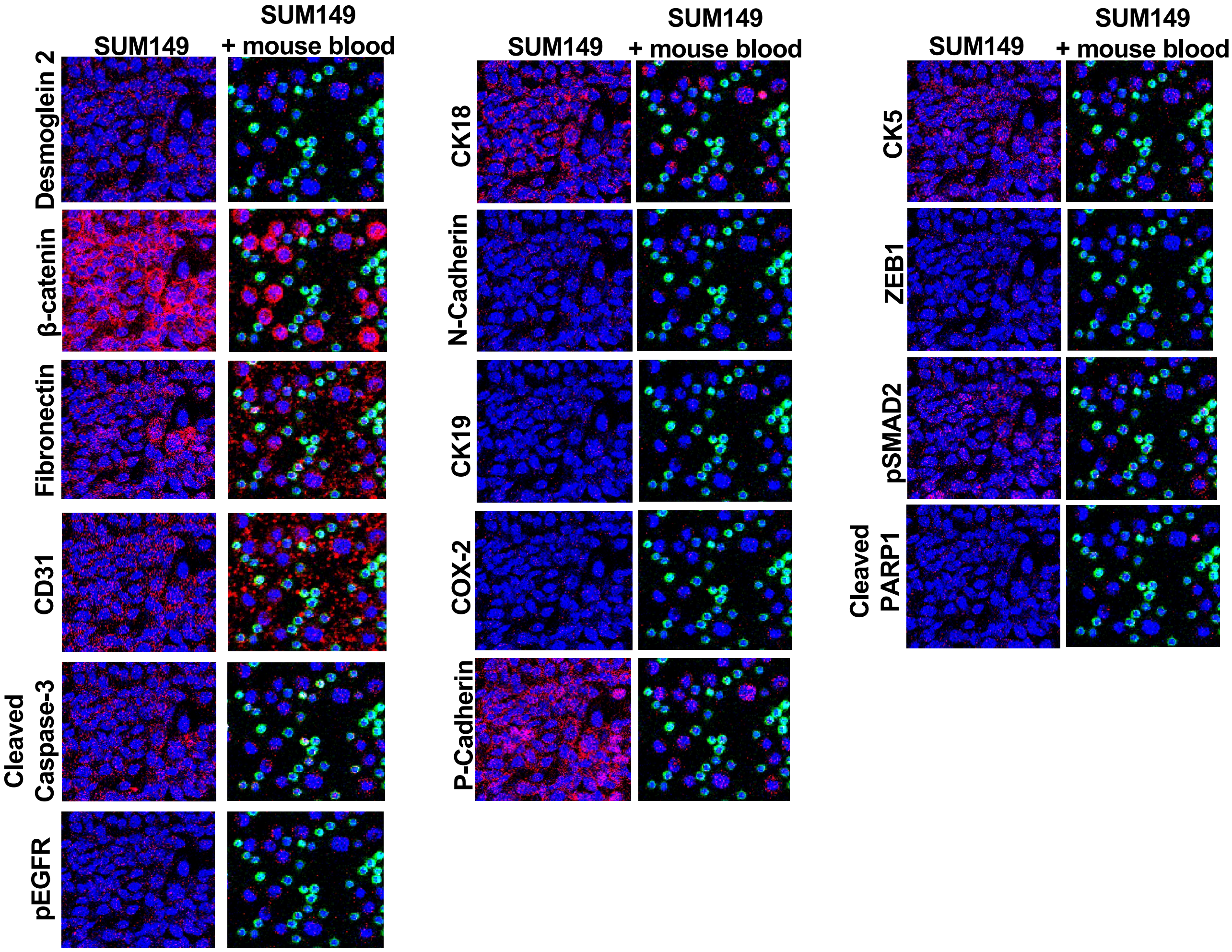

Supplementary Figure S1: Comparison of IMC signals by specific antibodies in SUM149 cells with and without admixed mouse blood. Representative images showing each target in red pseudo color, DNA in blue and CD45 in green.

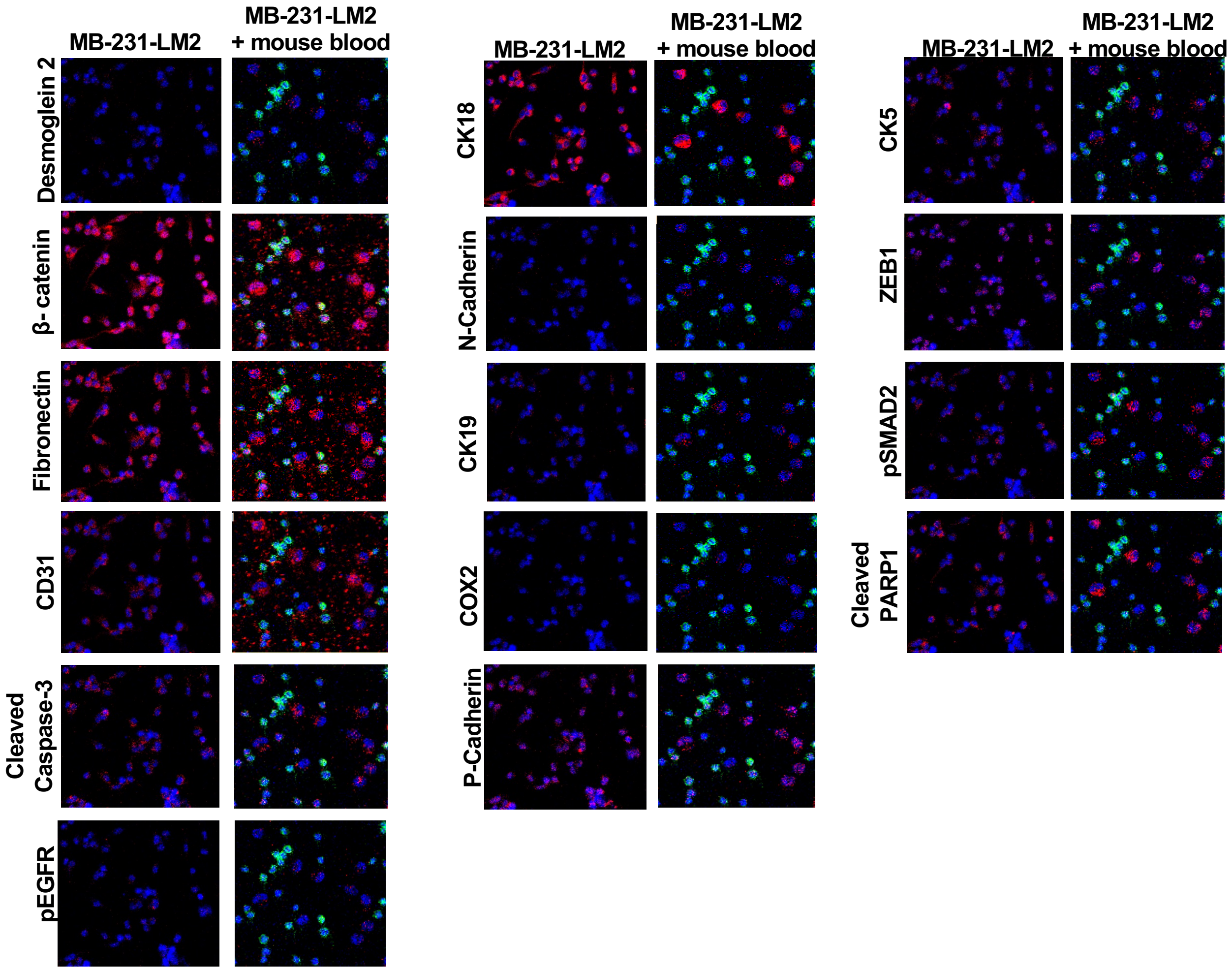

Supplementary Figure S2: Comparison of IMC signals by specific antibodies in MDA-MB-231-LM2 cells with and without admixed mouse blood. Representative images showing each target in red pseudo color, DNA in blue and CD45 in green.

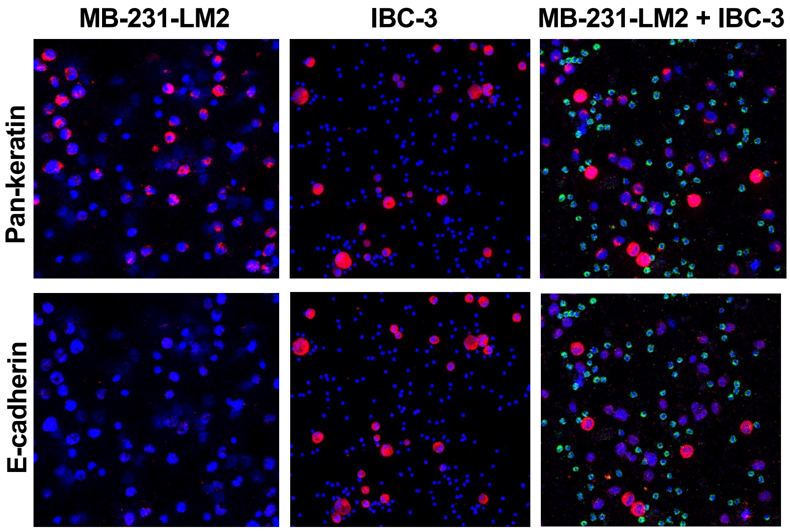

Supplementary Figure S3: Validation of E-cadherin antibody. Expression of PanCK and E-cadherin as detected by clone 24E10 in MDA-MB-231-LM2 and IBC-3 cells individually (CD45 signal was omitted) and spiked together into mouse blood (CD45+ staining in green).

A.

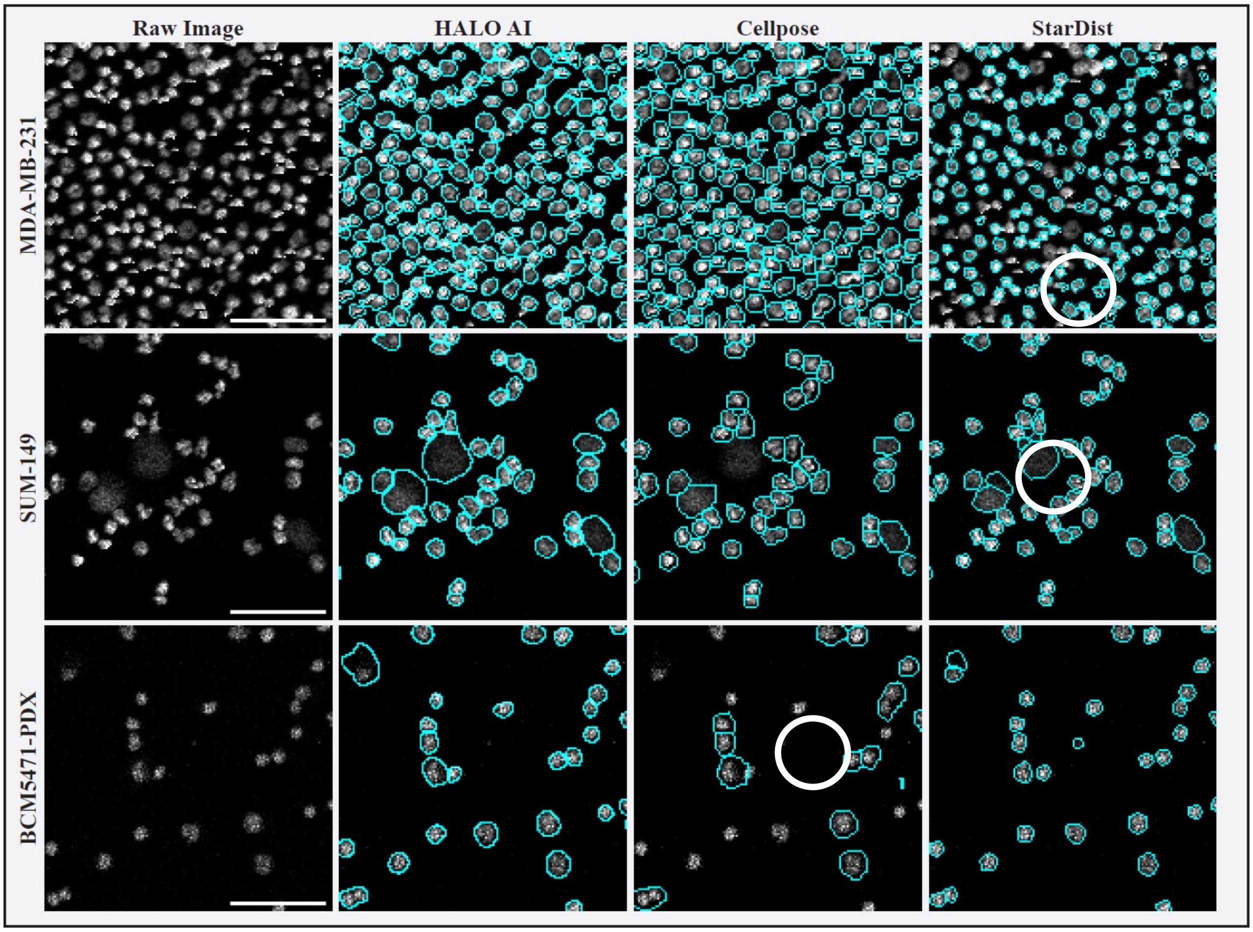

B.

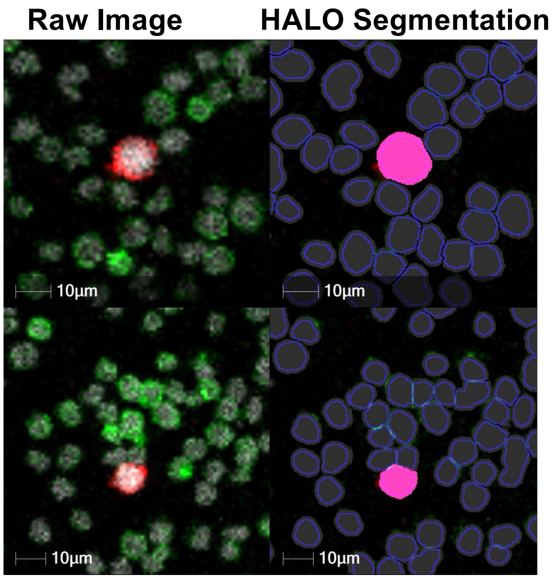

Supplementary Figure S4. Identification of CTCs by an automated and custom AI Halo cell segmentation classifiers. A) Nuclear segmentation of liquid biopsies from three tumor cell models across three segmentation algorithms, HALO AI, Cellpose, and StarDist (scale bars, 50µm). White circles indicate regions with errors. **B)** Example of HALO segmentation images derived from the raw data as I ndicated (DNA: white, PanKeratin: red, CD45: green).

Table S1: Antibodies used or tested for IMC in this study

Target	Category	Metal Tag	Clone	Vendor	Catalog Number	Concentration	Comment
beta-Catenin	Signaling	151Eu	196618	Biotechne	MAB13291-100	1 µg/ml	Non-specific signal
Caspase 3, cleaved	Cell death	172Yb	Asp175	Standard BioTools	3172027D	1 µg/ml	
CD31/PECAM-1	Endothelial	154Sm	390	Thermo Fisher	14-0311-85	1 µg/ml	Non-specific signal
CD44	Signaling	173Yb	156-3C11	CST	3570	0.5 µg/ml	
CD45	Mouse-specific	175Lu	30-F11	Standard BioTools	3175010B	1 µg/ml	
CEBPD	Signaling	149Sm	EPR23518-259	Abcam	ab270410	1 µg/ml	No signal
COX2	Signaling	160Gd	D5H5	CST	73315SF	1µg/ml	No signal
Cytokeratin 18	Luminal	155Gd	810811	Biotechne	MAB7619	0.5 µg/ml	
Cytokeratin 19	Luminal	159Tb	BA17	Biotechne	MAB3506	0.5 µg/ml	
Cytokeratin 5	Basal	163Dy	monoclonal	LS Bio	LS-C812437-100	1 µg/ml	
Cytokeratin 8	Luminal	152Sm	LP3K	Biotechne	MAB3165	0.5 µg/ml	
Desmoglein 2	Epithelial	146Nd	6D8	Biotechne	LS-C761566-100	1 µg/ml	weak signal
E-cadherin	Epithelial	148Nd	180224/24E10	Biotechne/CST	MAB18381/3195	0.5 µg/ml	No signal
E-cadherin	Epithelial	158Gd	24 E10	Standard BioTools	3158029D	1 µg/ml	
EGFR	Signaling	144Nd	AY13	Biolegend	352902	1 µg/ml	
EGFR (pY1068)	Signaling	162Dy	D7A5	CST	48576SF	1 µg/ml	
EpCAM	Epithelial	147Sm	9C4	Biolegend	324202	1 µg/ml	
Fibronectin	Mesenchymal	153Eu	EPR23110-46	Abcam	ab268022	1 µg/ml	
MHC Class II	Human-specific	154Sm	TDR31.1	LS Bio	LS-B6315-50	1 µg/ml	
N-cadherin	Mesenchymal	158Gd	CDH2/1573	LS Bio	LS-C761774-100	1 µg/ml	
Na/K ATPase	Human-specific	170Er	D4Y7E	CST	23565S	1 µg/ml	No signal
NOS2	Signaling	145Nd	4 E5	Novus	NBP2-22119	1 µg/ml	
NUMA1	Human-specific	141Pr	SPM300	LS Bio	LS-C390797-100	1 µg/ml	High background
PARP1, cleaved	Cell death	168Er	E51	Abcam	ab203467	1 µg/ml	
P-cadherin	Epithelial	161Dy	106020	Biotechne	MAB761-100	1 µg/ml	weak signal
Pan keratin	E/M	169Tm	Polyclonal	Thermo Fisher	26411-1-AP	0.33 µg/ml	
pan-Cytokeratin	Epithelial	148Nd	AE-1/ AE-3	Standard BioTools	3148022D	0.25 µg/ml	
SMAD2	Signaling	167 Er	EP784Y	Abcam	ab157371	1 µg/ml	
SMAD2 (pS467)	Signaling	165Ho	EPR23681-40	Abcam	ab280897	1 µg/ml	weak signal
Vimentin	Mesenchymal	150Nd	RV203	Abcam	ab8979	0.5 µg/ml	
ZEB1	Mesenchymal	164Dy	EPR17375	Abcam	ab228986	1 µg/ml	

Table S2: Comparison of the frequency of CTCs in blood collected by tail vein versus cardiac puncture.

Mouse I.D.	Cardiac	Tail vein	TV (mm^3)
SUM149 #1	12	n.d.	3704.8
SUM149 #2	2	n.d.	3269.3
SUM149 #3	0	n.d.	2572.5
SUM149 #4	4	1	2178.6
SUM149 #5	0	1	2384.8
SUM149 #6	1	0	2151.7
MB-231-LM2 #1	0	n.d.	2100
MB-231-LM2 #2	12	n.d.	2427.6
MB-231-LM2 #3	6	n.d.	1825.2
MB-231-LM2 #4	0	1	2112.0
MB-231-LM2 #5	6	2	3447.8
MB-231-LM2 #6	15	4	2788.2
BCM5471 #1	3	n.d.	2048.0
BCM5471 #2	0	n.d.	2427.6
BCM5471 #3	n.d.	3	2573.5
BCM5471 #4	37	2	1825.9
BCM5471 #5	10	16	2573.5
BCM5471 #6	18	6	2664.4

Manual count of the number of CTCs per 100 uL of blood collected from 6 mice each per tumor model (n.d., not done; TV, tumor volume).

Table S3: Threshold parameters for calling PanKeratin+ CTCs by HALO HighPlex FL module per tumor model and instrument.

Tumor model	Instrument	DNA3	PanKeratin*	CD45*
BCM5471	Hyperion+	≥ 7	≥ 1.5 (40%)	< 1.5 (25%)
BCM5471	Hyperion XTI	≥ 3	≥ 2.75 (35%)	< 1.5 (25%)
MB-231-LM2	Hyperion+	≥ 10	≥ 3 (45%)	< 1.5 (25%)
MB-231-LM2	Hyperion XTI	≥ 1.75	≥ 3 (50%)	< 1.5 (25%)
SUM149	Hyperion+	≥ 7	≥ 1.8 (40%)	< 1.5 (25%)
SUM149	Hyperion XTI	≥ 10	≥ 6 (50%)	< 1.5 (25%)

*Signal intensity thresholds and % completeness, i.e. percentage of the cell area covered by signal.

Table S4: Comparison of CTC counts by HALO compared to manual counts.

Mouse Model Sample#	Manual 1	Manual 2	HALO
SUM-149 #1	15	12	14
SUM-149 #2	2	2	5
SUM-149 #3	0	0	1
SUM-149 #4	1	1	0
SUM-149 #5	3	4	3
SUM-149 #6	0	0	0
SUM-149 #7	4	1	2
SUM-149 #8	0	0	1
SUM-149 #9	2	1	1
MB-231-LM2 #1	0	0	1
MB-231-LM2 #2	0	12	1
MB-231-LM2 #3	4	6	3
MB-231-LM2 #4	2	2	2
MB-231-LM2 #5	16	15	14
MB-231-LM2 #6	1	4	0
MB-231-LM2 #7	0	0	1
MB-231-LM2 #8	9	9	8
MB-231-LM2 #9	6	6	4
MB-231-LM2 #10	3	3	3
BCM-5471 #1	3	3	4
BCM-5471 #2	0	3	3
BCM-5471 #3	0	0	0
BCM-5471 #4	23	37	26
BCM-5471 #5	0	2	0
BCM-5471 #6	2	3	3
BCM-5471 #7	6	10	9
BCM-5471 #8	3	16	8
BCM-5471 #9	17	18	18
BCM-5471 #10	0	6	2

CTC frequency (#CTC/100uL blood) as assessed in the same ROI by two independent investigators and as determined by HALO.
